# Items of News and Gossip

**Published:** 1868-05-01

**Authors:** 


### *EDITORIAL.*

### *Items of News and Gossip.*

At a recent trial in the United States Court in this city, his Honor, Judge Drummond, sustained a witness in refusing to testify as an expert without having first received honorary fees therefor:

"The Court (Drummond) held that much injustice was probably done, and that, too, not unfrequently, to physicians and their patients, by taking them from their practice to compel them to remain from one to five hours upon the witness stand, that they might be examined as to all they knew or did not know upon a given subject. The whole matter of compelling a witness to testify he understood to be within the discretion of the Court, and, although he had the power to compel an answer, he did not think any exercise of his discretion would be required in this case. The Court further held that it was perfectly proper for parties employing physicians or other experts, to pay them fees before they came into court, and that such payment was morally and legally right, and not to the prejudice of the cause in which they might be called."

This is creditable to the Court, but it is a little funny, to say the least of it, that the witness who made the issue with the examining lawyer is a homœopathist, and, if the testimony in the particular case is to be believed, altogether *in*expert. Physicians called in these cases can control the *honoraria* if they choose to do so. No party to a case, properly defied, would venture an "attachment" to bring an expert into court.——An operator in the Medical College of a State across the lake, has initiated the practice of sending written invitations to prominent civilians ("laymen") in the village and country round about, to be present at his *cliniques* when any cutting is to be done. This may be *en regle* in Portland and Berkshire County, but we have heretofore been so obtuse as to think it expressly prohibited by the *Code of Ethics* adopted by the American Medical Association.——Professor Green, of the medical department of the University of Michigan, has resigned. It is rumored that A. B. P. proposes hereafter to occupy, "jointly and severally," the chairs of Practice of Medicine and Surgery in that somewhat dilapidated concern. This is said to be the real reason of his late visit to Chicago, to consult with the Apostle, and not, as previously reported, to get the latter's opinion on the propriety of holding on to his original position in Ann Arbor. It is whispered that the Apostle proposes to compromise, by adding a class of *Freshmen* to his educational schedule, especially for the accommodation of homœopathic nurslings. The latter can as well be supplied at the numerously appointed (and reappointed) Reform School in this city as in Michigan, and, meanwhile, save the Board of Regents an immensity of "bother." They have only to repeal their illiberal restriction of the location of the infinitesimal branch to that little but lively State. There is room enough in the southern suburbs of this city for immense attenuation. Let both parties hasten to do these things, and thus prove that a man *can* lie down with dogs and not get up covered with fleas.——And, speaking of fleas, it is said that the "Black Knight," whose prowess has so often saved the University of Michigan from a want of harmony, visited Detroit the other day to try and patch up an organ in that pleasant hamlet, of which he might become either a pipe or bellows. But the Detroit brotherhood thought him too scratchy for the one, and too windbroken for the other, whereupon he retreated to Ann Arbor again with a very large flea in each ear. We prophesy that he will stay in Ann Arbor, homœopathy or no homœopathy. We suggest to our friend Dr. Lewitt to furnish a Faculty for the proposed hybrid from his tanks of "pickles;" *Harmony* shall thus ever abound.——To the Journal box has come a letter most graphically addressed: "*To the Physician who thinks more of curing patients than having his own way.*" Enclosed was a handbill, offering the usual assurances and certificates of infallibility in the cure of scarlatina, diphtheria, etc., etc., all for ten dollars, cash in hand, with two years' credit for fifteen more. For this sum the author, claiming to be an M.D., offers to inform any physician of the method. We understand a large number of these circulars have been sent to the physicians of the North-west. This "confidence game" will scarcely win among the intelligent practitioners of this section. Meanwhile, if we had our "own way," we should lose patience long enough to compel the author of the handbill to swallow both it and his nostrum.——Professor Peaslee's last case of ovariotomy died on the fourth day, although apparently doing well until a few hours before death. "Commenced vomiting up dark green matter—vomited large quantities, and violently, and *collapsed in twenty minutes.*" Jacobi said the vomiting was *cerebral.* Hamilton does not like ovariotomy; J. R. Wood, ditto. Too uncertain; it is a kind of surgery you can tell nothing about, etc., etc.,—as we extract from a private letter from New York. The letter from our Heidelberg correspondent on this subject will attract attention. So far as present appearances go, the operation is speedily to become much less frequent, if not pass into desuetude.——Dr. Emmett, of New York, who probably stands unrivaled in this country as an operator in *uterine surgery*, opposes vehemently the operation of dilatation of the female urethra for the removal of stone. Our gossip says: "He makes a vesico-vaginal fistula. Such as *he makes* he has not the slightest trouble in closing—will often close themselves. He has been operating on a number of cases for painful, non-curable chronic cystitis, by making a vesico-vaginal fistula, and letting the bladder *rest a year!* That was new to me in the way of *surgical therapeutics.* Says there is not the slightest difficulty with the fistula. He makes it at a point where there will be *no loss of tissue.* Indeed, the parts coapt so well that the surgeon has to break up the adhesions once every twenty-four hours for quite a time, in order to get a fistule."——One of the Chicago Sanitary Inspectors gives us a case wherein small pox being present in a family, they refused to have any one of their three unprotected children vaccinated, because their physician said, if they should happen to take the small pox, the addition of the vaccine disease would add greatly to the danger. So strong was their faith in this statement, that the three children were spirited away for fear the Board of Health would seize and vaccinate them *nolens volens.* At present writing, one of the three is already down with variola.——The Board of Health are entitled to great credit for the persistent efforts they have put forth to secure general vaccination of the children in the city.——The "*Hahnemann Medical College*" building, in this city, has been seized by the officers of the United States government, it having been used as a cover to a *vinegar factory*, in which the shrewd detectives found a concealed still, with all its appurtenances. It is claimed, however, that the still was only in use for preparing "mother tinctures" and attenuations. There is much consternation in homœopathic circles in the city over this *mesalliance* of vinegar, whisky, law, and infinitesimals. The Faculty of the College refuse to testify as *experts* in the litigation which is to follow, unless allowed honorary fees. It is hoped these will be properly liquidated. ——The New York *Medical Gazette* attributes the establishment of the homœopathic department of the University of Michigan to the attempts made to remove the previously existing medical department to Detroit. Of our own knowledge, these attempts were abandoned years ago, and had nothing to do with the affair whatever. The recent defeat of the prohibitory liquor law in Michigan, the $2 00 tax on whisky, and the recent seizure of the Hahnemann College in this city, show that it is a well concocted plan of the "whisky ring" to establish a huge manufactory of contraband spirits, to be run at the expense of the Peninsular State.

